# Asymptomatic bacteriuria in recurrent UTI – to treat or not to treat

**DOI:** 10.3205/id000035

**Published:** 2017-12-28

**Authors:** Tommaso Cai, Riccardo Bartoletti

**Affiliations:** 1 Department of Urology, Santa Chiara Regional Hospital, Trento, Italy; 2 Department of Translational Research and New Technologies, University of Pisa, Italy

## Abstract

Asymptomatic bacteriuria (ABU) is a common clinical condition that often leads to unnecessary antimicrobial use. The reduction of antibiotic overuse for ABU is consequently an important issue for antimicrobial stewardship to reduce the emergence of multidrug resistant strains. In the clinical setting we have an important issue that requires special attention: the role of ABU in women affected by recurrent urinary tract infections (rUTIs). In everyday clinical practice, young women affected by rUTI show after antibiotic treatment asymptomatic periods associated sometimes with or without bacteriuria. Although it is not recommended, the majority of women with ABU is treated with poor results and occasionally a selection of multidrug-resistant bacteria can be observed. Recent studies demonstrated that ABU should not be treated in young women affected by rUTI, because it may play even a protective role in preventing symptomatic episodes, particularly when Enterococcus faecalis has been isolated. Moreover, ABU treatment is associated with a higher occurrence of antibiotic-resistant bacteria, indicating that ABU treatment in women with rUTIs is even potentially dangerous.

## Summary of recommendations

1. Asymptomatic bacteriuria (ABU) is a bacterial colonization, but not an infection that requires treatment.
2. ABU is generally common in women affected by recurrent UTIs, specially after antibiotic treatment.
3. ABU has a protective role in preventing symptomatic recurrences, particularly when *Enterococcus faecalis* has been isolated.
4. ABU treatment is associated with a higher occurrence of antibiotic-resistant bacteria.
5. ABU treatment in women with rUTIs is therefore potentially dangerous.

## Introduction

This review addresses the role and management of asymptomatic bacteriuria (ABU) in women affected particularly by recurrent urinary tract infections (rUTI). ABU in pregnant women, in men, in children, after transplantation, before urological surgery etc. are not addressed in this review.

## Methods

This review incorporates the 2016 Guidelines on Urological Infections of the European Association of Urology (EAU) and the latest Infectious Diseases Society of America Guideline for the management of ABU [1], [2]. Moreover, a systematic literature search was performed in Medline, Cochrane, and Embase. The following keywords have been used: asymptomatic bacteriuria and recurrent urinary tract infection. The limitations included adult with age over 18 years, clinical studies, English and peer reviewed. A total of 161 publications were identified and screened by title and abstract. All publications about pregnancy, transplantation, prophylaxis, and children were excluded. Finally, 14 papers were included in this review. The studies were rated according to the level of evidence (LoE) and the grade of recommendation (GoR) using ICUD standards [3]. 

## Definition, context and clinical application

### Microbiological definition

An episode of ABU was defined as the presence of at least 10^5^ Colony Forming Unit (CFU) of uropathogenic bacteria per milliliter in two consecutive voided urine specimens of a midstream urine specimen obtained from an asymptomatic woman on a routine scheduled visit [1].

### Clinical setting

ABU is a bacterial colonization that does not indicate an infection and is generally present in 3% to 5% of young women [4], [5]. Although ABU is more common in patients with diabetes mellitus and elderly persons, its treatment is generally recommended only in pregnant women and at the pre-operative evaluation before surgical procedures [1], [2], [5]. Regardless of these recommendations, overuse of antibiotics in ABU treatment is common. In fact, about one-third of ABU were over-treated contrary to the guidelines, with important negative consequences on public health [6], [7], [8]. In everyday clinical practice, young sexually active women affected by rUTI show, after antibiotic treatment, an asymptomatic period associated sometimes with or without bacteriuria [9]. Although it is not recommended, the majority of women with ABU is treated with poor results and with a high risk of selecting multidrug resistance pathogens [10]. Recently, Cai et al. even demonstrated a protective effect of spontaneously developed ABU in women with rUTIs without other associated risk factors [9]. However, some authors stated that occasionally the eradication of a strain considered the causative agent of recurrent episodes of UTI may be justified [11]. Against this background, the question arises: What is the role of ABU treatment in women with rUTI?

## What is the role of ABU treatment in women with recurrent urinary tract infections?

Recently, Cai et al. showed, in a randomized clinical trial and in a prospective cohort study, four important findings [9]:

1. treatment of ABU is associated with a higher probability to develop symptomatic recurrence rate;
2. treatment of ABU is associated with a modification of the isolated bacterial strains;
3. the presence of ABU in patients affected by rUTI, without any associated risk factor, has a protective role in development of subsequent symptomatic UTI, particularly when *Enterococcus faecalis* has been found;
4. treatment of ABU is associated with a higher prevalence of antibiotic-resistant bacteria [10].

The change in isolated bacteria from the urinary tract after antibiotic therapy is well known and known to be dangerous in several cases. Beerepoot et al. demonstrated that oral administration of low dose antibiotics for the prevention of UTIs could cause ecological disturbances in normal intestinal microflora, while promoting the emergence of antimicrobial-resistant strains [12]. Moreover, several authors demonstrated that antibiotic therapy is able to disturb the ecological balance in the colon tract and to suppress the normal microflora [12], [13]. The ecological effects of antibacterial agents on the human microflora should be the main reason of the negative effect of antibiotic therapy in women affected by rUTIs with ABU. The normal bacterial intestinal flora represents an extremely important defense mechanism, which effectively interferes with the establishment of many important enteric pathogens [14]. It is well known that mechanisms by which microorganisms suppress the growth of other microorganisms include modification of bile acids, stimulation of peristalsis, induction of immunologic responses, depletion of essential substrates from the environment, competition for attachment sites, creation of restrictive physiologic environments, and elaboration of antibiotic-like substances [13], [14]. For example, it has been demonstrated that normal bacterial intestinal flora is able to stimulate the production of secretory IgA, an antibody class unique to the mucosae [15]. In this sense, the presence of IgA in the intestinal lumen should be considered a primitive front line defense against induction of autoimmunity and invasion by microbial pathogens [16]. Components of the intestinal microbial flora also interact synergistically in the induction of disease or the utilization of substrate. In this sense, we can hypothesize that* E. faecalis* should be an extremely important defense mechanism, which effectively interferes with the establishment of many important enteric pathogens, such as *E. coli* [9], [10]. Finally, in two randomized controlled trials on the prevention of recurrent UTI in non-hospitalized premenopausal and postmenopausal women [Non-antibiotic prophylaxis for recurrent urinary tract infections’ (NAPRUTI) study], Beerepoot and co-workers found that the predictive values of the susceptibility pattern of the ABU strain, based on resistance prevalence at baseline, were 76%, except in the case of nitrofurantoin and amoxicillin-clavulanic acid-resistance [17]. Moreover, they found that the susceptibility pattern of *E. coli* strains isolated during the month before a symptomatic *E. coli* UTI can be used to make informed choices for empirical antibiotic treatment in this patient population [17].

### Role of ABU in older women with recurrent UTI

It is well known that the incidence of ABU increases from 3.5% in the general population to 16% to 18% in women older than 70 years and some longitudinal studies report that it affects 50% of older women [18]. In one longitudinal prospective series of ambulatory older adults, patterns of bacteriuria observed in urine samples obtained at 6-month intervals revealed that more than 30% of patients had spontaneously resolving bacteriuria and another 30% who initially did not have bacteriuria subsequently developed it [18], [19]. On the other hand, even if UTI in older women can be a serious problem, due to the risk of upper urinary tract infections, several randomized controlled trials found that 25% to 50% of women presenting with UTI symptoms will have recovered in 1 week without using antibiotics [20], [21]. In this sense, delaying antibiotic treatment while evaluating a symptomatic UTI generally does not lead to adverse outcomes [18].

Most authors therefore suggest to delay antibiotic treatment while conducting further evaluation when the diagnosis of symptomatic UTI is in doubt in older women and offer supportive treatment such as increased fluid intake. In fact, spontaneous symptom improvement occurs in 50% of community-dwelling non-catheterized women who delay antibiotic treatment [22], [23]. Moreover, in 1994, Abrutyn and co-workers demonstrated that reinfection rates (1.67 vs 0.87 per patient-year of follow-up), adverse antimicrobial drug effects, and isolation of increasingly resistant organisms occur more commonly in ABU therapy vs non-therapy groups [24]. 

## Conclusions

Evaluation of the role of ABU in women affected by rUTIs is a key point in order to optimize antibiotic usage and to prevent an increased rate of resistant bacteria. In line with the latest studies, all physicians should be aware of these findings and avoid antibiotic treatment of ABU in women with rUTI. 

## Notes

This article is also to be published as a chapter of the Living Handbook „Urogenital Infections and Inflammations“ [25].

## Conflict of interest

The authors declare that they have no competing interests.
